# Genes Involved in Sex Pheromone Discrimination in *Drosophila melanogaster* and Their Background-Dependent Effect

**DOI:** 10.1371/journal.pone.0030799

**Published:** 2012-01-23

**Authors:** Benjamin Houot, Stéphane Fraichard, Ralph J. Greenspan, Jean-François Ferveur

**Affiliations:** 1 Centre des Sciences du Goût et de l'Alimentation, UMR6265 Centre National de la Recherche Scientifique, Université de Bourgogne, Dijon, France; 2 Kavli Institute for Brain and Mind, University of California San Diego, La Jolla, California United States of America; Washington University Medical School, United States of America

## Abstract

Mate choice is based on the comparison of the sensory quality of potential mating partners, and sex pheromones play an important role in this process. In *Drosophila melanogaster*, contact pheromones differ between male and female in their content and in their effects on male courtship, both inhibitory and stimulatory. To investigate the genetic basis of sex pheromone discrimination, we experimentally selected males showing either a higher or lower ability to discriminate sex pheromones over 20 generations. This experimental selection was carried out in parallel on two different genetic backgrounds: wild-type and *desat1* mutant, in which parental males showed high and low sex pheromone discrimination ability respectively. Male perception of male and female pheromones was separately affected during the process of selection. A comparison of transcriptomic activity between high and low discrimination lines revealed genes not only that varied according to the starting genetic background, but varied reciprocally. Mutants in two of these genes, *Shaker* and *quick-to-court*, were capable of producing similar effects on discrimination on their own, in some instances mimicking the selected lines, in others not. This suggests that discrimination of sex pheromones depends on genes whose activity is sensitive to genetic context and provides a rare, genetically defined example of the phenomenon known as “allele flips,” in which interactions have reciprocal effects on different genetic backgrounds.

## Introduction

The role of contact pheromones in the courtship and mate discrimination of *Drosophila melanogaster* is now well established [1], [2], [3]. Natural variation in the ability of males to discriminate sexual partners has also been demonstrated, and shown to depend in large measure on pheromonal responses [4], [5]. Analysis of induced mutants has shown a major role for the *desat1* locus in both pheromone production and mate discrimination [6], [7], where it affects each of those processes independently [8].

Laboratory selection experiments in *Drosophila* have demonstrated the ease with which fruit fly behavior can be altered and the abundance of the reservoir of natural variation capable of producing such modifications [9]. With the advent of whole-genome assays, it has become possible to begin identifying the genetic and molecular correlates of selection-induced changes in behavior, many of which have been shown to have phenotypic consequences [10]. Selection for alterations in courtship behavior have been an integral component of these studies [9], [11].

Given the importance of pheromone production and response in *D. melanogaster* and its evolutionary plasticity, we set out to probe the molecular nature and mechanisms of genetic variation in mate discrimination. To this end, we have carried out laboratory selection for increased or decreased mate discrimination starting from two different genetic backgrounds: a wild-type strain and a *desat1* mutant strain where males respectively showed high and low ability to discriminate sex pheromones. We decided to use a mutant impaired for discrimination in order to see whether we could further change this response, particularly in the direction of improving it, in keeping with a history of such experiments in this organism [9]. We also chose this strategy to identify other genes that may be involved, given the many recent findings on the wide range of genes capable of affecting any phenotype [10]. Our results show that genetic background can strongly influence the roles played by individual genes in behavior, even to the point of having diametrically opposite effects.

## Results and Discussion

**Figure 1:**
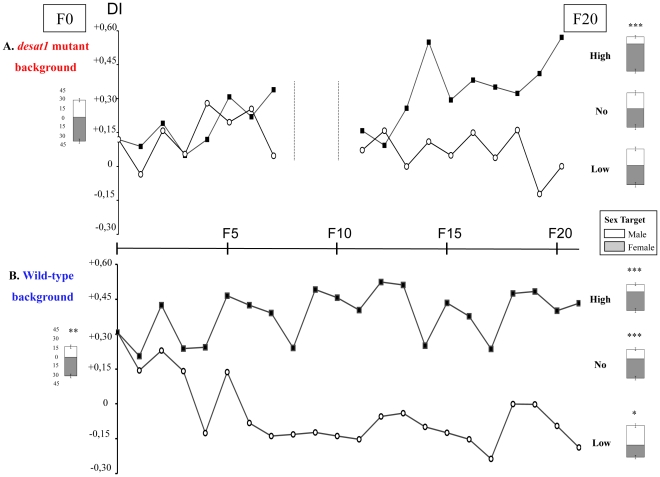
Selection for male discrimination of sex pheromones in two genetic backgrounds. Experimental selection was carried out in a *desat1* mutant background (A) and in a wild-type background (B). At each generation, and until generation 21 (F21), single tester males were selected for their ability to discriminate wild-type male and female targets simultaneously presented. Histograms indicate the mean (±sem) of the courtship index directed towards target males (empty bars, CIm) and target females (filled bars; CIf). CIm and CIf are shown for both parental strains (*desat1* and wild-type; left) and for selected lines with high (“High”; right) and low discrimination (“Low”) and for unselected lines (“No”). Data shown for each generation represent the discrimination index (DI = [CIf−CIm]/[CIf+CIm]) which was calculated on “High” lines (filled squares), and “Low” lines (empty circles). The level of significance of discrimination, assayed with a paired *t*-test is indicated above bars: *** *P*<0.001; ** *P*<0.001; * *P*<0.05. N≥60.

To determine the ability of single tester males to court and discriminate sex partners, we measured the courtship intensity that they directed toward both female and male flies presented simultaneously. This allowed us to measure the courtship intensity (measured as courtship index, CI) towards a target fly of either sex (CIf = towards a female; CIm = towards a male), and calculate the ability of tester males to distinguish between the two sex targets. The ability of male flies to discriminate between the sexes was determined by the comparison of both CIf and CIm for each genotype and condition (Figure 1; discrimination index, DI = [CIf−CIm]/[CIm+CIf]). Unless specified, tests were carried out with immobilized target flies under red light, in which flies are effectively blind. This makes the behavioral effect of pheromones more pronounced, due to the lack of other contributing visual cues [3]. For the sake of clarity, DIs are shown at each generation whereas the CIf/CIm comparison are only shown at the initial and final generations.

Before initiating the experimental selection, we measured the discrimination in parental males of the *desat1* mutant and wild-type strains (F0; Figure 1). Mutant males showed no significant CIf/CIm difference (*P* = ns), indicating no significant difference in their courtship of either the female or the male target, and their DI was slightly positive (+0.14; Figure 1A). In contrast, CIf in wild-type males was significantly higher than CIm (t = 3.063; *P* = 0.0027), and their DI was positive (+0.31; Figure 1B).

### Discrimination Behavior in Selection Lines

Performance results for the selected lines were pooled from the scores of four parallel sub-lines in each case (see Material and Methods). The first selection experiment carried out with *desat1* mutant flies produced significant differences between lines selected for high discrimination (Figure 1A; filled squares) and low discrimination (empty circles) after seven successive generations of selection (F7; for technical reasons, the selection procedure was relaxed during the F8, F9 and F10 generations, and then reinitiated at F11, see Material and Methods). The comparison of individual DIs between low and high lines revealed significant differences only at F15 (Kw = 4.659; *P* = 0.031), F20 and F21 (Kw = 14.665 and14.743; *P* = 0.0001). At F20, the CIf/CIm intra-strain comparison revealed a significant difference in discrimination for high lines (t = 8.366; *P*<0.001), but no discrimination (*P* = ns) either for the low lines or the unselected lines. This indicates that the process of selecting males with increased discrimination in the mutant lines required at least 10 generations of selection.

The second selection experiment carried out on flies with a wild-type background revealed a significant difference for individual DIs between the high and low lines after only 4 generations (Figure 1B; F4; Kw = 4.876; *P* = 0.027). Between F6 and F21, this significant difference persisted and increased to reach a higher level of significance at F21 (Kw = 17.139; *P*<0.0001). At F21, the CIf/CIm difference was also highly significant for both high and unselected lines (*P*<0.001) whereas it was reduced in the low discrimination lines (*P*<0.05). The divergence of behavior so early in a selection experiment may be indicative of the presence of some strong effect variants in the population [9], [10], or of a behavior that is not so firmly anchored genetically.

### Male and Female Pheromone Discrimination in Selection Lines

**Figure 2:**
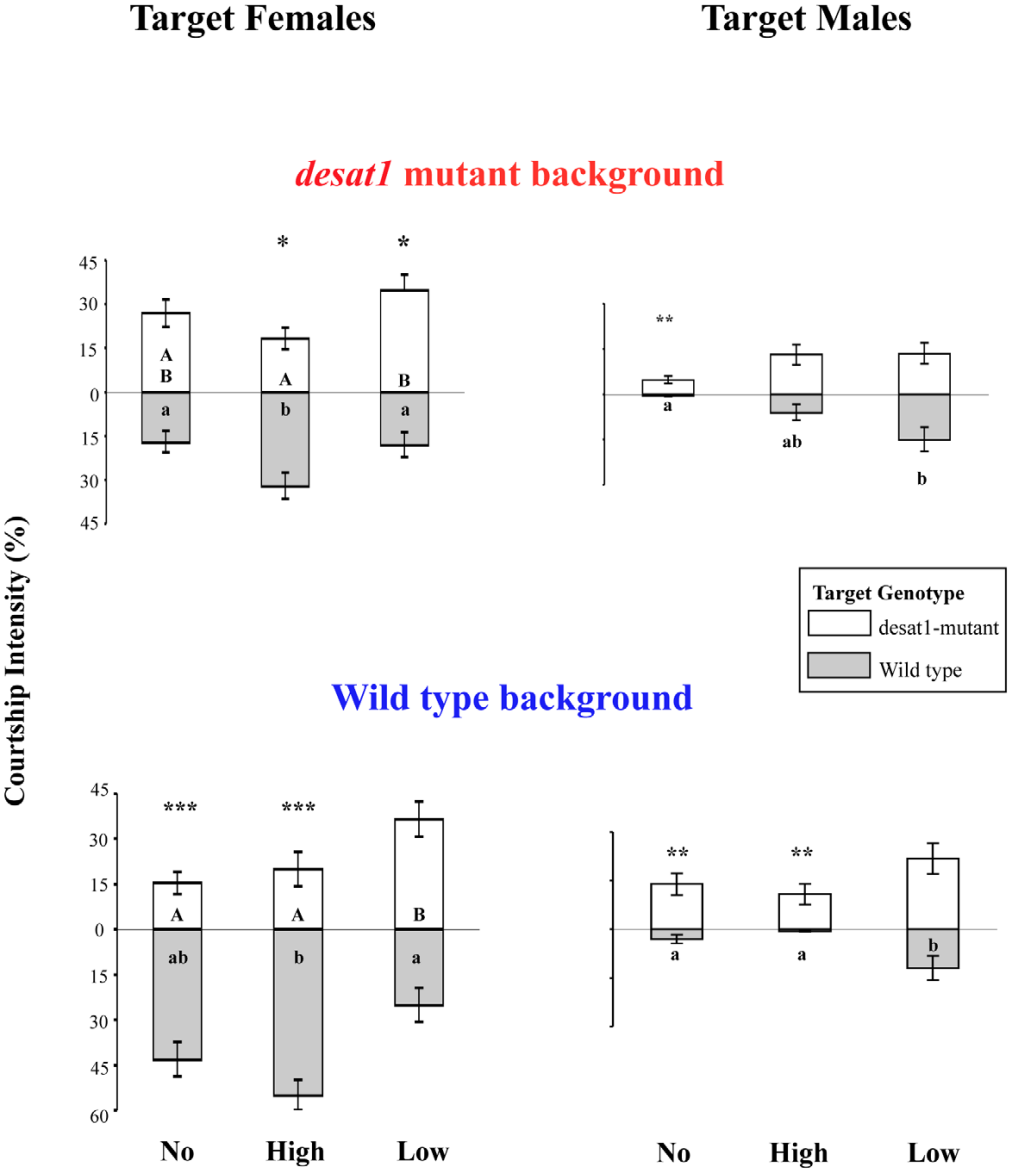
The selection process separately affects discrimination of female and male pheromones. At F20 and F21, males from the differently selected lines (No, High, Low) with the *desat1* mutant (top) and wild-type backgrounds (bottom) were simultaneously presented to a pair of same-sex partner flies of *desat1* (empty bars) and wild-type genotypes (filled bars). Mutant *desat1* males and females produce a reduced level of sex pheromones compared to same-sex wild-type flies. Histograms represent the mean courtship index (±sem) directed toward each target. The significance level of discrimination is indicated above each bar (t test). Different letters inside the bars (capital for *desat1* target, lower case for wild-type target) indicate significant difference relatively to each target genotype (ANOVA with multiple pairwise comparaison and Bonferroni post-hoc tests). For all other information, please refer to Fig. 1. N≥45.

Since the altered discrimination could be caused by altered perception of pheromones produced by either sex or both sexes, we measured the selected males' ability to respond to pheromones of each sex separately (Figure 2). Males of each selected line and of the unselected line were presented to a pair of same-sex target flies, one from the *desat1* mutant strain and one from the wild-type strain: *desat1* mutant females and males produce much less of the contact pheromones (such as 7,11-heptacosadiene and 7-tricosene, respectively [7]) than wild-type flies. Note that the selection procedure did not affect the hydrocarbon content in males of the different lines (Table S1).

Same-sex tests were performed with F20 *desat1*-background males. With target females, unselected males showed no discrimination, whereas the two groups of selected males showed different preferences: high males slightly preferred the wild-type target female and low males preferred the *desat1* target female (*P*<0.05 for either sex partner). This variation is likely caused by the significantly higher CI shown by low lines toward *desat1* target females — and to their lower CI to wild-type target females — as compared to high lines (*P*<0.05). This indicates that wild-type female pheromones induced excitatory responses in the high and inhibitory responses in the low males, respectively. In contrast, the relatively high discrimination of male pheromones shown by unselected males (*P*<0.01) disappeared in both high and low selected lines (*P* = ns) while the CI directed towards wild-type target males significantly increased in low males (*P*<0.05). This indicates that low males have lost their ability to detect and/or respond to wild-type male pheromones.

In wild-type background males, same-sex tests were performed at the F21 generation of selection. With target females, unselected males always strongly preferred wild-type target females (*P*<0.001). In high lines, the discrimination ability was also high (*P*<0.001) whereas low lines showed no discrimination (*P* = ns). Moreover, low males increased their response to *desat1* target females — and decreased their response to wild-type target females — compared to high lines (*P*<0.05). This suggests that low males are unable to perceive wild-type female pheromones. With male targets, high males show higher discrimination than low males (respectively, *P*<0.01 and *P* = ns). This effect was likely caused by the increased CI of wild-type target males by low lines (compared to high lines; *P*<0.05). These data indicate that low males are less repulsed than the two other males by wild-type male pheromones. The relative differences in response shown by selected males of the two backgrounds are thus generally consistent.

The present experiment, based on the ability of individual males to discriminate between a choice of two sensory cues allowed us to focus more specifically on pheromonal cues. With both genetic backgrounds, our selection process separately altered male response to male *vs.* female pheromones. A similar experiment revealed that male flies successively used olfactory and gustatory cues to choose a sex partner [12]. This process may involve peripheral sensory neurons as well as dedicated brain centers [13], [14], where *desat1* could be expressed [6], [8].

### Identification of Altered Genes

Next, to identify some of the genes involved in this variation of discrimination ability, we performed microarray analysis on *desat1* mutant background males from high, low and unselected lines and compared separately the RNA extracted from the head and from the rest of the body. Note that we did not carry out microarray analysis on the wild-type background strain because we wanted to emphasize the extremes of behavior, knowing that in short-term selection experiments (i.e., anything less than 50 generations), gene expression differences are not large [10]. We focused our attention on candidate genes showing variation in the head but not in the rest of the body and retained those showing the greatest variation between lines with the highest *P*-value. For the best candidate genes, the microarray variation was verified using Real-time PCR (q-PCR; Table S2). Among 13 microarray candidates in *desat1* males, only 4 genes showed significant q-PCR variation: *Nf1*, *Shaker* (*Sh*) and *CG4187* expression significantly increased in high lines (*P*<0.002−0.001) whereas *quick-to-court* expression increased in low lines (*qtc*; *P*<0.001).

**Figure 3:**
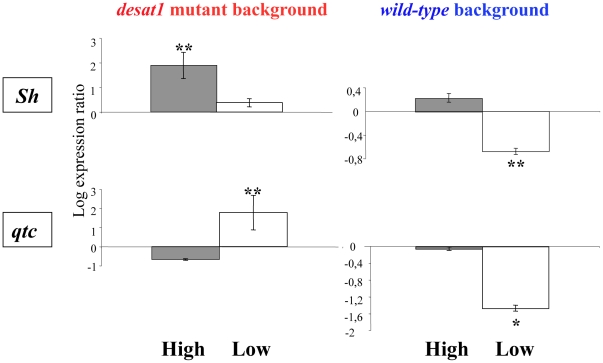
Gene expression activity depends both on the direction of selection and genetic background. The level of transcriptional activity was measured by q-PCR for the *Shaker* (*Sh*, top) and *quick-to-court* (*qtc*, bottom) genes in two genetic backgrounds: *desat1* (left) and wild-type (right). Ordinate denotes to the log ratio of expression in the heads of 5-days old adult males in lines selected for high (“High”, filled bars) and low (“Low”, empty bars) sex pheromone discrimination relative to unselected lines (“No”; corresponding to “0”). Statistics indicate the significance of the variation between selected and unselected lines. For all other information, please refer to Fig. 1.

Using similar q-PCR conditions, we probed the 13 candidate genes in wild-type background selected lines (Table S2), and found that *Sh* and *qtc* expression was significantly decreased in low lines (*P* = 0.018 and 0.002, respectively). Therefore, the two genes varied differentially with respect to genetic background and selection line (Figure 3). *qtc* expression level varied reciprocally between low lines of the two selected strains: it increased in mutant and decreased in wild-type background. *Sh* also varied between backgrounds, but in this case it increased in mutant high lines and decreased in wild-type low lines.

*Shaker* and *quick-to-court* have both been previously shown to have courtship effects. *Shaker* —a part of voltage-dependent potassium ion channel— is defective in the plasticity associated with male courtship [15], which could be related to the altered processing of gustatory inputs in the central nervous system [16]. *quick-to-court*, a gene encoding a predicted protein with coiled-coil domains, was isolated as an insertion (enhancer-trap) expressed in the antennal olfactory organ and elsewhere in the brain, and shown to have enhanced courtship of females by males [17].

### Functional Tests of Altered Genes in the Two Backgrounds

**Figure 4:**
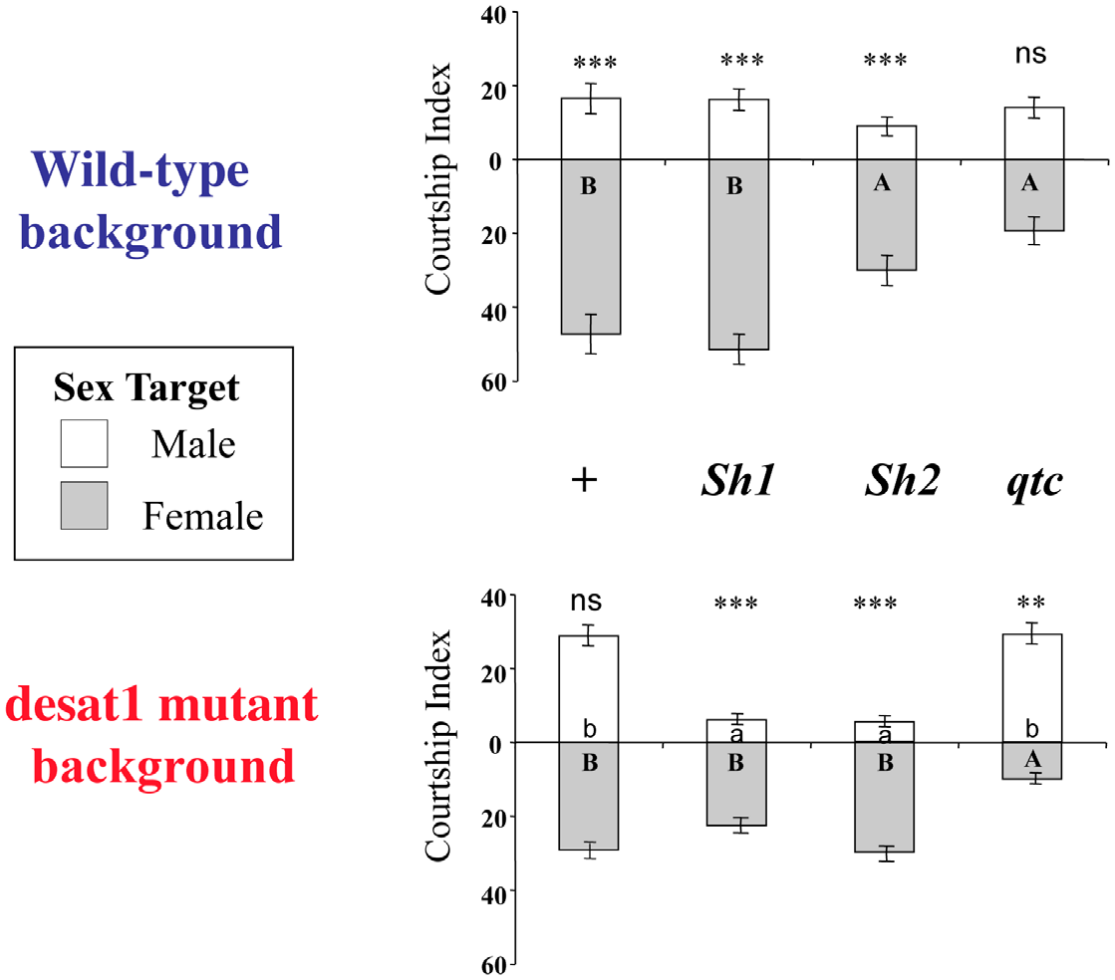
Behavioral effect of genetic mutations in two genetic backgrounds. The effect of mutations of the two genes *Shaker* (*Sh*) and *quick-to-court* (*qtc*) on males' ability to discriminate wild-type target males (empty bars) and females (filled bars) was measured. Histograms represent the intensity of courtship directed towards each sex-target. The effect of the *qtc* mutation and of two *Sh* mutations (*Sh1*, *Sh2*) was measured in wild-type (top) and *desat1* backgrounds (bottom). The significance of the difference in discrimination ability is represented above each bar. The different letters inside the bars (capital for female targets, lower case for male target) indicate significant differences relative to each target. N≥45. For all other information and statistics, refer to Figs. 1 and 2.

To validate the behavioral function of *qtc* and *Sh*, we genetically manipulated the two genes and measured the discrimination of manipulated males. First, we tested male discrimination in *qtc* and *Sh* mutants on the wild-type background (Figure 4, top). The two *Sh* mutants (*Sh1* and *Sh2*) showed a high level of male discrimination (*P*<0.001), whereas *qtc* mutant males failed to discriminate sex targets (*P* = ns). Moreover, the intensity of courtship towards target females (CIf) was significantly reduced in both *Sh2* and *qtc* mutant males indicating their decreased perception and/or response to wild-type female pheromones. Given the reciprocal variation noted for *qtc* expression and the similar variation for *Sh* expression, we tested the interaction effect of each mutation in the *desat1* mutant background (Figure 4, bottom). Males doubly mutant for *desat1* and *qtc* showed significant discrimination ability (*P*<0.001), but in the opposite direction to normal discrimination: they spend more time courting target males than target females, and their courtship of females is the lowest for any of our genotypes (CIm = 26; CIf = 12.8). This result is consistent with the response shown by low *desat1*-background males to same-sex flies (Figure 2). These selected males showed a decreased response to wild-type female pheromones and increased response to wild-type male pheromones. In males doubly mutant for *desat1* and either *Sh* mutation, the presence of the *Sh* mutation restored the ability to discriminate (*P*<0.001) that was otherwise lacking in the *desat1* mutant alone. Moreover, these males showed less courtship of other males than any of our other genotypes. Singly and doubly mutant females showed no obvious variation of their mating pattern (data not shown).

Behavioral selection for altered mate discrimination in different *D. melanogaster* genetic backgrounds has demonstrated that the same gene can display reciprocal effects in different genetic backgrounds. Whereas the effect of genetic background on behavior has been shown many times (reviewed by [18]), our study reveals that it can interact with a given gene in opposite ways to alter a phenotype. Specifically, we have shown not only that the two genes *Shaker* and *quick-to-court* both show altered expression in the high *vs.* low discrimination lines on both the wild-type and *desat1* backgrounds, but that they also diverge in the direction of the alterations. Tests of induced mutations in these genes demonstrates their capability of affecting the discrimination phenotype, though not always in the direction predicted from the expression level.

That is, the induced *qtc* mutant shows low discrimination on a wild-type background, just as the low selected line on that same background has reduced expression of the *qtc* gene. When we tested double mutants of *qtc* in combination with *desat1*, we found that it produces a high level of discrimination, but in the wrong direction. *qtc;desat1* double mutants show the strongest preference for courting other males and the lowest level of courtship of females of any genotype tested. From the stand-point of discrimination *per se*, this result says that the *qtc* mutant exerts opposing phenotypic effects on the two different genetic backgrounds.

In contrast, both *Sh* mutants on the wild-type background continue to show high levels of discrimination, even though the low selected line on that background is low in *Sh* expression. This suggests that the *Sh* difference on that background is either irrelevant, or else acts epistatically with something else in that selected background. When we tested the effect of double mutants of *Sh* in combination with *desat1*, we found that *Sh* mutations restore the normal discrimination that is otherwise lacking in *desat1* mutants. This runs counter to the *Sh* expression levels in the *desat1* selected background, where high levels of *Sh* correlate with high discrimination. Again, this suggests that the *Sh* alterations due to selection may be irrelevant to the phenotype.

### Targeting candidate genes in specific tissues

**Figure 5:**
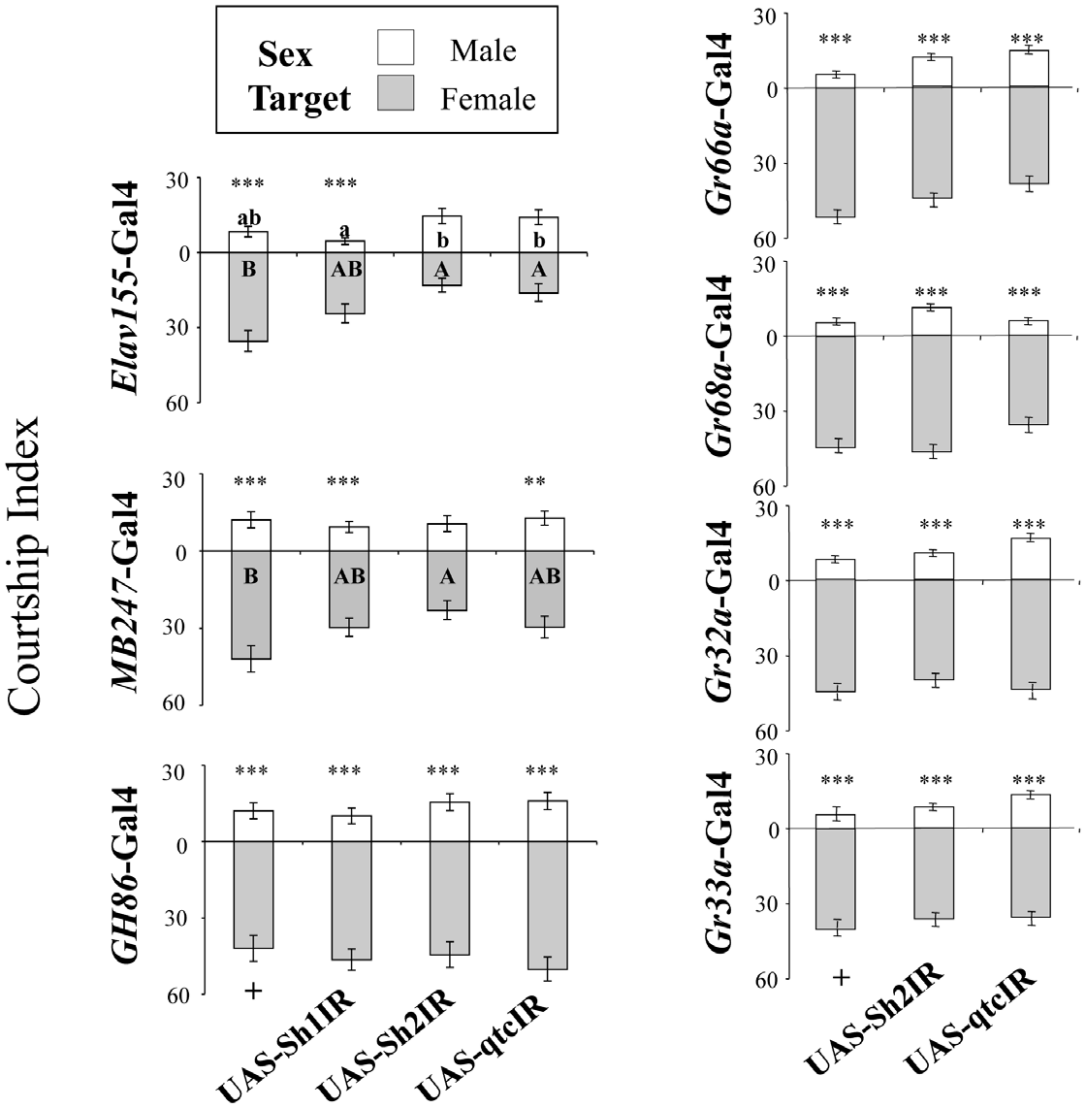
Behavioral effect of the RNAi of the two “discrimination genes” targeted in different subsets of the nervous system. The effect of targeted RNAi expression of the two genes *Shaker* (*Sh*) and *quick-to-court* (*qtc*) on males' ability to discriminate wild-type target males (empty bars) and females (filled bars) was measured. Histograms represent the intensity of courtship directed towards each sex-target. Two RNAi transgenes against *Sh* (*UAS-Sh1IR*, *UAS-Sh2IR*) and one RNAi transgene against *qtc* (*UAS-qtcIR*) were targeted pan-neurally (by *Elav155-Gal4*), in the mushroom bodies (*MB247-Gal4*), in most peripheral neural sensory neurons (*GH86-Gal4*; left). *UAS-Sh2IR* and *UAS-qtcIR* were also targeted in peripheral taste neurons expressing the Gr66a, Gr68a, Gr32a or Gr33a receptors (*Gr66a-Gal4*, *Gr68a-Gal4*, *Gr32a-Gal4*, *Gr33a-Gal4*; right). N≥32. For statistics, see Figs. 2 and 4.

To identify the tissues expressing either *qtc* or *Sh* and involved in male discrimination, we targeted RNAi reporter transgenes to affect expression of either gene in various portions of the nervous system. First, a Gal4 driver transgene targeting most adult neurons (*Elav155-Gal4*; Figure 5) driving a RNAi transgene and affecting each gene (*UAS-Sh2IR*; *UAS-qtcIR*) allowed us to completely abolish sex discrimination. In males expressing these two RNAi constructs (nearly) pan-neurally, the CIf was significantly decreased, and the CIm significantly increased, compared to control males. Using q-PCR, we found that the expression level of both genes was significantly decreased in the male heads of both genotypes: 1/2.65 in *Elav155-Gal4>UAS-qtcIR* and 1/2.0 in *Elav155-Gal4>UAS-Sh2IR* (Figure S1). A driver transgene expressed in the mushroom bodies (*MB247-Gal4*) also allowed us to reduce or even abolish male discrimination in males expressing these two RNAi constructs. However, targeting the RNAi transgenes to most chemosensory peripheral neurons (*GH146-Gal4*) did not affect male discrimination. We also targeted both *UAS-Sh2IR* and *UAS-qtcIR* transgenes to different subsets of peripheral taste neurons known to be involved in sex pheromone response (Gr66a [19]; Gr68a [20]; Gr32a [21], [22]; Gr33a [23]; Figure 5). All of the *Gr-Gal4>UAS-RNAi* males showed high levels of discrimination (*P*<0.001), indicating that the expression of neither *qtc* nor of *Sh* is required in these taste neurons for proper pheromone discrimination. Since *desat1* also affects the level of sex pheromones, we measured the amount of these compounds in all transgenic flies (Table S1). All double mutant males (homozygous for the *desat1* mutation) showed a profile similar to *desat1* mutant males, whereas all other single mutant and transgenic males showed a pheromonal profile similar to that of wild-type flies. Moreover, the effect of these genes might be sex-specific since no alteration of female mating behavior was detected in the mutant and RNAi targeted strains.

Previous studies reported that the genetic alteration of peripheral taste neurons expressing Gr66a, Gr32a and Gr33a affected male response to a male inhibitory pheromone [19], [22], [23], [24]. Similarly, the alteration of CheB42a, a putative pheromone ligand in the sheath cells surrounding Gr68a-Gal4-expressing neurons [25], decreased male response to female pheromone [26]. However, our data (Figure 5) suggest that neither *qtc* nor *Sh* are necessary in these taste neurons. Male discrimination of sex partners is thought to be based on the olfactory perception of volatile pheromones such as *cis*-Vaccenyl-acetate, which is processed in the lateral protocerebrum [14], a brain center also implicated in the activation of male courtship [27], [28]. Complete neural expression of the specific RNAi of both *Sh* and *qtc* completely affected male sex pheromone discrimination, but when expression was restricted in the brain, only the RNAi of *Sh* could also abolish discrimination. Our experiment did not allow us precisely to target the tissues where the RNAi of *qtc* should be targeted to abolish male discrimination, as the detailed expression pattern of this gene has not been mapped. But our results do indicate that the expression of the two genes in different neural tissues is necessary for normal pheromonal discrimination.

In conclusion, the finding that gene interactions vary combinatorially and with genetic background is not at all surprising and has been seen many times before (e.g. [10], [18]). Examples of genes exerting opposite effects on phenotype, as appears to be the case for *qtc* in our experiment, are not uncommon. Among the earliest well defined examples are some of the homeotic developmental genes found in *Drosophila* and in *C. elegans* (reviewed in [29]). In these cases, opposite developmental transformations are produced by gain *vs.* loss of function alleles. More recently, association studies in human disease have turned up many cases of “allele flips,” in which the same marker appears associated with the phenotype under study, but positively in some populations and negatively in others. Examples have been found in association studies of asthma, autism, late-onset Alzheimer's, and schizophrenia [30]; (reviewed in [31], [32]). In the absence of any experimental tests, the likely explanation is that there is some kind of interaction between the locus in question and some other locus or loci in the genetic background, as appears to be the case in our study for the phenotype of the *qtc* mutant on the two different backgrounds.

“Allele flips” provide us with yet another example of the profound influence of genetic context on gene interactions, and thus on phenotype. Such effects have been seen in opposing effects of QTLs affecting life span in males vs. females [33]. This has been found to be true even on well defined backgrounds, as in our previous study showing that the interactions among a set of genes which are part of a common phenotypic network is substantially altered when the genotype at a single locus is altered [34]. Many of these alterations in the alternative backgrounds produced “allele-flip”-like switches in phenotype. A similarly potent influence of context was shown in a study that compared the phenotype of a developmental mutant when placed on a series of undefined, but markedly different, genetic backgrounds [35]. The mutant phenotype was found to vary all the way from wild-type to that of a null allele across this range of backgrounds. Our system, by permitting a direct experimental test, allowed us to mimic the genetic background difference with defined mutations and confirm such an interaction, thus uncovering a small portion of the results of selection. As succinctly put by Lewontin at the end of *The Genetic Basis of Evolutionary Change*, “Context and interaction are of the essence.” [36].

## Materials and Methods

### Strains and crosses

All *D. melanogaster* strains were raised on yeast/cornmeal/agar medium and kept at 24±0,5°C with 65±5% humidity on a 12 L: 12 D cycle. Dijon2000 (DIJ) is the wild type strain used as control [8]. The *desat1* mutant strain contains a PGal4 transposon inserted in the regulatory region of this gene [7] and shows reduced production of sex pheromones and altered male discrimination of sex pheromones [6], [7]. Before starting the experiment on the *desat1* strain, the *desat1* mutation was outcrossed during 5 generations in a white-eyed DIJ strain. Crosses were performed using standard techniques and genetic tools [37].

The *Shaker* and *quick-to-court* mutant stocks were obtained from the Bloomington Drosophila Stock Center (*Sh1* = *w [1118] Mi{ET1}Sh [MB00560]*, #22837; *Sh2* = *w [1118] Mi{ET1}Sh [MB02366]*, #24181; *qtc* = w1118; P{XP}qtcd00941, #19155). These mutations were tested in homozygous flies with a DIJ wild-type background, and in the *desat1* background (for the *qtc* and *Sh2* mutations). The double mutant *desat1*; *qtc* strain was built following a 5-generations procedure using the double balancer strain *CyO*; *TM3* (#250, Bloomington Stock Center). The double mutant *desat1*; *Sh2* strain was built following a 3-generations procedure using the *TM3*,*Sb* balancer strain and screening the presence of *Sh* with the leg-shaking phenotype under anesthesia.

The *Elav155-Gal4* strain was generously given by Prof. A.K. Guo (Institute of Neurosciences, Shanghai), the *MB247-Gal4* and *GH-146-Gal4* were kindly provided by Prof. R.F. Stocker (University of Fribourg), and *Gr66a*-, *Gr68a*-, *Gr32a*- and *Gr33a-Gal4* by Profs. Kristin Scott, Hubert Amrein and Craig Montell. The *UAS-RNAi* transgenes were purchased from the VDRC [38]. All of these transgenics were mated with the DIJ wild-type strain to test for any dominant effect (controls). *UAS-RNAi* transgenics were mated with each of the seven Gal4 drivers to assess their combined effect on behavior.

### Behavior and experimental selection

All flies were isolated 0–4 h after eclosion under CO_2_ anaesthesia. Tester male flies (i.e. those whose sexual response to target flies was measured) were held individually in fresh glass food vials for 5 days before testing. Target flies were similarly treated but they were held in groups of five for the same period. All tests were performed in a room at 24±0.5°C with 65±5% humidity, between 9am and noon when flies show a peak of sexual activity. Tester males were individually aspirated (without anaesthesia) under a watch glass used as a courtship observation chamber (1.6 cm^3^). After 5 min to allow the tester male to habituate to the chamber, the two control target flies (a male and a female) were introduced and the observation period started.

To characterize male discrimination of sex pheromones, we measured the proportion of time spent by tester males in actively courting (wing vibration, licking and attempted copulation; total = courtship index; CI) each target. For each male, we obtained two values corresponding to the CI directed to the male (CIm), and to the female target (CIf; [8]). Note that the total CI (CIf+CIm) can vary between 0 and 100. Tests were carried out under a dim red light (25 W with a Kodak Safe-light filter n°1) to remove all visual stimuli [39] and target flies were decapitated to remove most acoustic and behavioral signals [40]. Within each strain, the difference between CIm and CIf was measured with a Student's t-test. The CI towards each target was tested between genotypes with a ANOVA completed by a multiple pairwise comparaison using Bonferroni post-hoc tests.

The experimental selection was carried on two different genetic backgrounds (*desat1*, wild-type) during two successive years. At each generation we selected between 60 and 80 males (for the 4 lines pooled) for the high line and the same number for low lines. They were mated to sibling females from the same generation. Numbers were similar for the two backgrounds.

In the first year, the selection carried out with the *desat1* background (interrupted between F8 to F11 for unavoidable technical reasons, in order to be able to continue with no change in procedures) was maintained until F20. The second year, selection was carried out with the wild type background (without any interruption) until F21. Twenty generations has generally been found to be a reasonable length of time to obtain changes in phenotype and gene expression in behavioral selections [9], [10]. The selection was initially carried out on parental lines resulting from the pooling of 20 isofemale lines raised separately. At each generation, individual males were tested for their discrimination ability: those showing either the highest or the lowest CIf/CIm ratio —but still directing courtship towards the two target flies— were kept to induce the «high» and «low» discrimination lines, respectively. Basically, “high lines” selected males showed a CIf≥2 CIm and “low lines” selected males had a CIm≥2CIf. Males showing the most extreme CIf/CIm differences were kept, and those with CIf+CI m<10 were excluded to avoid a biased CIf/CIm comparison. For each selection direction and genotype, we established four parallel selection sub-lines, selecting only the single male with the highest or lowest ratio. These selected males were mated with sibling females from the same generation and subline to yield the next generation.The four sublines showing high male discrimination and the four sublines showing low males discrimination were separately selected during these experiments. In parallel, four sublines with non-selected flies were transferred and kept in similar conditions. The behavioral and transcriptomic data shown here are pooled data from the sublines, a step that was necessary in order to have sufficient material and number of samples for statistical analysis. It also allowed us to pool the effects and thus to emphasize the major effects.

Two statistical tests were performed to evaluate male ability to discriminate. The intrastrain discrimination was assayed with a paired *t*-test between individual CIf and CIm values. For the sake of clarity, and to follow the variation of male discrimination between generations, we designed the discrimination index, DI = [CIf−CIm]/[CIm+CIf]. The comparison of DIs between low and high selected lines was assayed at each generation with a Kruskall-Wallis test. Note that only flies with [CIm+CIf]>10 were retained to avoid the bias caused by low CIs.

### Microarrays and q-PCR

For microarrays and q-PCR experiments, total RNA was extracted from homogenized heads or from bodies by the Trizol method (GIBCO BRL) and treated with RNase-free DNase to avoid contamination by genomic DNA [41]. Total RNA (2 µg) was reverse transcribed with the iScript cDNA Synthesis Kit (Biorad). For microarrays, 5 µg RNA from at least 4 biological replicates were obtained separately from the head and the rest of the body (thoraces+abdomens). RNA samples were hybridized to Affymetrix Drosophila 2.0 microarrays by the UCSD GeneChip Core. Raw data are provided in Table S3.

Quantative PCR reactions were performed with the IQ SYBR Green supermix (Biorad) in a thermal cycler (MyIQ, Biorad) according to the procedure recommended by the manufacturer. The qPCR reaction was done in a volume of 20 µl, by 40 cycles (95°C for 30 sec, TM °C for 30 sec and 72°C for 30 sec), preceded by 3 min denaturation step at 98°C and followed by a 1 min elongation step at 72°C. TM of the hybridization step depends on the primer pair used. Generally, we used a TM of 60°C for *qtc* (*qtc*Forward: GATTTGGCACAGCGTCAAC; *qtc*Reverse: GCGTATGTTCTCCAACTCGTC), *Shaker* (*Shaker*Forward: GAGGTGCCTGACA TCACAGA; *Shaker*Reverse: TGCGAGGAACCTGACAGTTA), and control actine5C (*Act60*Forward:TAACAAATTCAAGGCGTGAAA;*Act60*Reverse: TTCAGTCGGTTTATTCCAGTCA), Each reaction was performed in triplicate and the mean of the three independent biological replicates was calculated. All results were normalized to the *Actin5C* mRNA level.

Significant differences in transcript levels ratio between control and sample strain (body and head) were detected with the Relative Expression Software Tool (REST, REST-MCS beta software version 2 [42]) where the iteration number was fixed at 2000. This test is based on the probability of an effect as large as that observed under the null hypothesis (no effect of the treatment), using a randomization test (Pair Wise Fixed Reallocation Randomisation Test© [43]).

## Supporting Information

Figure S1 **q-PCR in RNAi strains.** RNA levels were measured in the heads of control (empty bars) and *Elav155-Gal4>UAS-RNAi* males (filled bars). When targeted by *Elav155-Gal4*, the two RNAi transgenes (*UAS-qtcIR*, top and *UAS-Sh2IR*, bottom) significantly (*: p<0.05) decreased the expression of *qtc* (1/2.65) and *Sh* (1/2.00), respectively. These data were obtained with 9 biological replicates.
(PDF)

Table S1 **Levels of the principal sex pheromones in manipulated males.** Data shown corrrespond to the mean (±sem; in ng) for the principal cuticular hydrocarbons in males of various strains. These compounds are: 7-tricosene (7-T), n-tricosane (23Lin), methyl-tetracosane (25Br), 7-pentacosene (7-P), n-pentacosane (25Lin), methyl-hexacosane (27Br), n-heptacosane (27Lin), methyl-octacosane (29Br). We also show the sum of all CHs (ΣCHs). From top to bottom, strains correspond to the wild-type (Dijon) and the mutant desat1 strains, to the high and low selected lines in the desat1 and in the wild-type backgrounds, to the qtc, Sh1, Sh2 mutations in the wild-type and in the desat1 backgrounds. N≥15.
(PDF)

Table S2 **Transcriptional variation of genes in lines selected for high/low discrimination in two genetic backgrounds.** The genes listed in the left column, initially detected by microarrays in the desat1 mutant background, were retained on the basis of high P-value in RNA extracted from the head vs. RNA extracted from the rest of the body. The variation of their transcriptional activity (x = increase; : = decrease) was measured by q-PCR in the desat1 (left) and wild-type (right) genetic backgrounds in males of the High and Low selected lines, relative to the level found in unselected lines of respective background. The levels were transformed on a log scale and the probability (P) of a significant variation indicated (ns = non significant).
(PDF)

Table S3 **Original microarray data.** Raw data for results in Table S2, from Affymetrix Drosophila 2.0 arrays on labeled RNA extracts from heads (He) and bodies (Bo) from unselected (Un), high (Hi) and low (Lo) lines. Each value is marked as “Present” (P) or “Absent” (A) based on Affymetrix' standard “detection above background” software analysis (http://media.affymetrix.com/support/technical/whitepapers/exon_background_correction_whitepaper.pdf).
(XLS)
