# Supplementary figures and images for: Genes Involved in Sex Pheromone Discrimination in Drosophila melanogaster and Their Background-Dependent Effect
Source: PLoS One. 2012 Jan 23;7(1):e30799. doi: 10.1371/journal.pone.0030799 (PMC3264623; doi:10.1371/journal.pone.0030799)

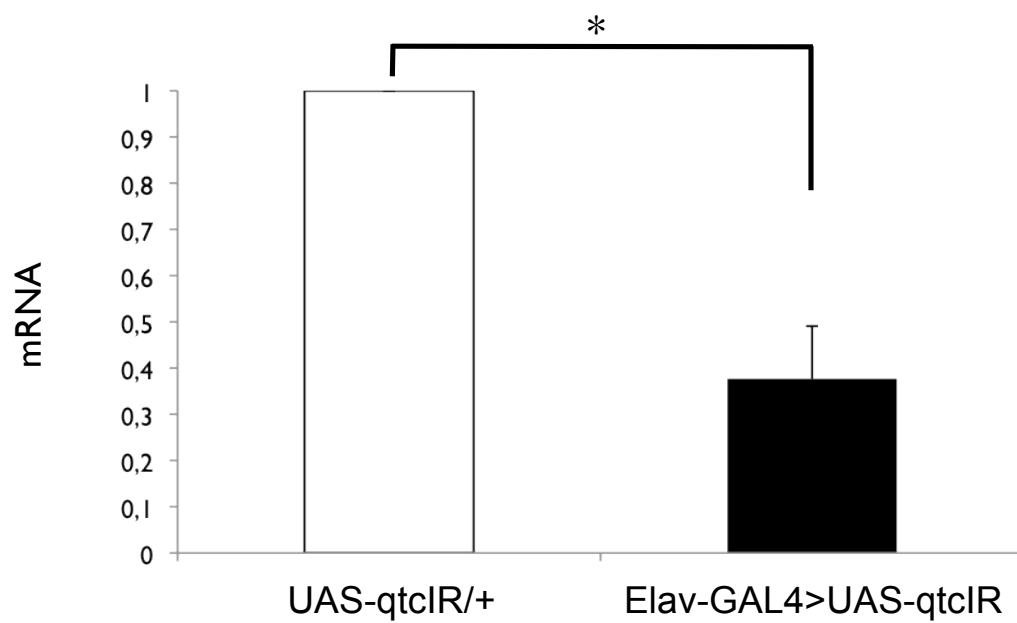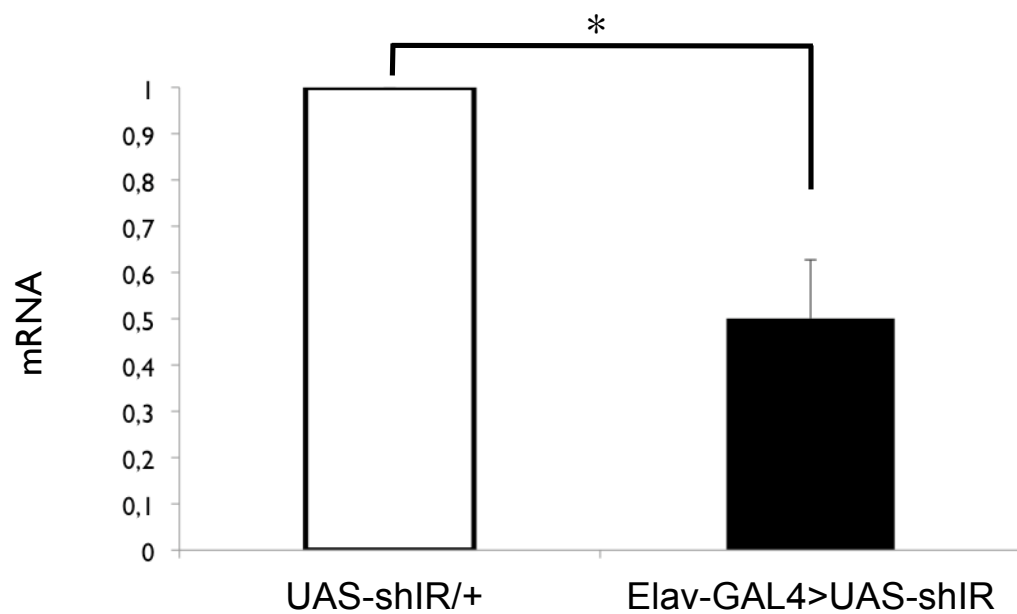

Supplement: Figure S1 — q-PCR in RNAi strains. RNA levels were measured in the heads of control (empty bars) and Elav155-Gal4>UAS-RNAi males (filled bars). When targeted by Elav155-Gal4, the two RNAi transgenes (UAS-qtcIR, top and UAS-Sh2IR, bottom) significantly (*: p<0.05) decreased the expression of qtc (1/2.65) and Sh (1/2.00), respectively. These data were obtained with 9 biological replicates. (PDF) [file pone.0030799.s001.pdf]
