# Supplementary material for: Genes Involved in Sex Pheromone Discrimination in Drosophila melanogaster and Their Background-Dependent Effect
Source: PLoS One. 2012 Jan 23;7(1):e30799. doi: 10.1371/journal.pone.0030799 (PMC3264623; doi:10.1371/journal.pone.0030799)
Supplement: Table S1 — Levels of the principal sex pheromones in manipulated males. Data shown corrrespond to the mean (±sem; in ng) for the principal cuticular hydrocarbons in males of various strains. These compounds are: 7-tricosene (7-T), n-tricosane (23Lin), methyl-tetracosane (25Br), 7-pentacosene (7-P), n-pentacosane (25Lin), methyl-hexacosane (27Br), n-heptacosane (27Lin), methyl-octacosane (29Br). We also show the sum of all CHs (ΣCHs). From top to bottom, strains correspond to the wild-type (Dijon) and the mutant desat1 strains, to the high and low selected lines in the desat1 and in the wild-type backgrounds, to the qtc, Sh1, Sh2 mutations in the wild-type and in the desat1 backgrounds. N≥15. (PDF) [file pone.0030799.s002.pdf]

**Hydrocarbons**<br><br>Genotype	**7-T**<br>(ng)	**23Lin**<br>(ng)	**25Br**<br>(ng)	**7-P**<br>(ng)	**25Lin**<br>(ng)	**27Br**<br>(ng)	**27Lin**<br>(ng)	**29Br**<br>(ng)	**∑CHs**<br>(ng)
Wild-type (Dijon)	991 ±54	287 ±11	156 ±6	112 ±7	44 ±3	134 ±5	15 ±1	71 ±3	1941 ±80
desat1 mutant	171 ±13	1720 ±105	307 ±16	50 ±3	384 ±39	260 ±12	101 ±7	117 ±6	3216 ±147
High Discrim. (*desat1*)	134 ±11	1962 ±237	296 ±15	40 ±2	431 ±55	254 ±17	108 ±13	122 ±10	3453 ±346
Low Discrim. (*desat1*)	104 ±5	1956 ±91	318 ±26	68 ±21	462 ±21	262 ±9	106 ±5	109 ±5	3466 ±137
High Discrim. (Dijon)	1073 ±47	276 ±12	150 ±10	140 ±13	47 ±3	146 ±5	20 ±5	80 ±3	2029 ±83
Low Discrim. (Dijon)	1239 ±152	331 ±34	287 ±114	168 ±13	61 ±6	147 ±7	20 ±2	86 ±3	2465 ±257
qtc mutant	704 ±129	180 ±32	116 ±18	204 ±30	30 ±4	170 ±11	14 ±1	91 ±4	1646 ±121
Sh1 mutant	592 ±71	157 ±7	83 ±10	157 ±17	31 ±2	142 ±8	12 ±2	81 ±6	1428 ±83
Sh2 mutant	551 ±39	152 ±7	68 ±5	184 ±23	24 ±1	157 ±11	12 ±1	99 ±7	1356 ±65
qtc/desat1 double mutant	150 ±18	1215 ±111	249 ±24	15 ±3	246 ±15	199 ±24	71 ±5	112 ±13	2351 ±208
Sh1/desat1 double mutant	114 ±7	1281 ±94	314 ±20	33 ±2	238 ±31	456 ±25	125 ±9	174 ±11	2869 ±171
Sh2/desat1 double mutant	84 ±8	1292 ±83	287 ±25	39 ±6	283 ±18	487 ±30	129 ±8	180 ±10	2869 ±156
