# Supplementary material for: Genes Involved in Sex Pheromone Discrimination in Drosophila melanogaster and Their Background-Dependent Effect
Source: PLoS One. 2012 Jan 23;7(1):e30799. doi: 10.1371/journal.pone.0030799 (PMC3264623; doi:10.1371/journal.pone.0030799)
Supplement: Table S2 — Transcriptional variation of genes in lines selected for high/low discrimination in two genetic backgrounds. The genes listed in the left column, initially detected by microarrays in the desat1 mutant background, were retained on the basis of high P-value in RNA extracted from the head vs. RNA extracted from the rest of the body. The variation of their transcriptional activity (x = increase; : = decrease) was measured by q-PCR in the desat1 (left) and wild-type (right) genetic backgrounds in males of the High and Low selected lines, relative to the level found in unselected lines of respective background. The levels were transformed on a log scale and the probability (P) of a significant variation indicated (ns = non significant). (PDF) [file pone.0030799.s003.pdf]

	**desat1 mutant Background**				**Wild type Background**			
Gene	High Line		Low Line		High Line		Low Line	
	Factor	*p*	Factor	*p*	Factor	*p*	Factor	*p*
Black	x0.63	*ns*	x1.19	*ns*	: 0.98	*ns*	x1.11	*ns*
Cpr49Ag	x0.22	*ns*	x2.23	*ns*	: 0.08	*ns*	x0.18	*ns*
Cyp4s3	: 0.29	*ns*	: 0.54	*ns*	: 0.12	*ns*	x0.26	*ns*
Cyp9c1	: 0,86	*ns*	x0.27	*ns*	x0.44	*ns*	x0.19	*ns*
Nf1	x2.23	*0.002*	x0.14	*ns*	x0.38	*ns*	x0.01	*ns*
pHCl	x0.99	*ns*	x1.01	*ns*	x0.33	*ns*	x0.30	*ns*
Qtc	: 1.34	*ns*	x3.54	*0.001*	: 0.06	*ns*	: 1.47	*0,002*
Shaker	x1.89	*0.002*	x0.38	*ns*	x0.23	*ns*	: 0.68	*0,018*
CG1492	x1.01	*ns*	x0.98	*ns*	x0.12	*ns*	: 0.19	*ns*
CG2893	x0.12	*ns*	x0.42	*ns*	x0.29	*ns*	x0.12	*ns*
CG4187	x2.25	*0.001*	x0.26	*ns*	: 0.52	*ns*	: 0.61	*ns*
CG4330	: 0.38	*ns*	: 1.88	*ns*	: 0.07	*ns*	x0.44	*ns*
CG18646	x0.08	*ns*	: 1.15	*ns*	: 0.39	*ns*	: 1.12	*0,041*
